# Scoping review of deep learning research illuminates artificial intelligence chasm in otolaryngology-head and neck surgery

**DOI:** 10.1038/s41746-025-01693-0

**Published:** 2025-05-10

**Authors:** George S. Liu, Soraya Fereydooni, Melissa Chaehyun Lee, Srinidhi Polkampally, Jeffrey Huynh, Sravya Kuchibhotla, Mihir M. Shah, Noel F. Ayoub, Robson Capasso, Michael T. Chang, Philip C. Doyle, F. Christopher Holsinger, Zara M. Patel, Jon-Paul Pepper, C. Kwang Sung, Francis X. Creighton, Nikolas H. Blevins, Konstantina M. Stankovic

**Affiliations:** 1 Department of Otolaryngology–Head and Neck Surgery, Stanford University, Stanford, CA USA; 2 Department of Otolaryngology–Head and Neck Surgery, Johns Hopkins University, Baltimore, MD USA

**Keywords:** Health care, Machine learning

## Abstract

Clinical validation studies are important to translate artificial intelligence (AI) technology in healthcare but may be underperformed in Otolaryngology - Head & Neck Surgery (OHNS). This scoping review examined deep learning publications in OHNS between 1996 and 2023. Searches on MEDLINE, EMBASE, and Web of Science databases identified 3236 articles of which 444 met inclusion criteria. Publications increased exponentially from 2012–2022 across 48 countries and were most concentrated in otology and neurotology (28%), most targeted extending health care provider capabilities (56%), and most used image input data (55%) and convolutional neural network models (63%). Strikingly, nearly all studies (99.3%) were in silico, proof of concept early-stage studies. Three (0.7%) studies conducted offline validation and zero (0%) clinical validation, illuminating the “AI chasm” in OHNS. Recommendations to cross this chasm include focusing on low complexity and low risk tasks, adhering to reporting guidelines, and prioritizing clinical translation studies.

## Introduction

Artificial intelligence (AI) technology is poised to enhance care delivery by physicians in otolaryngology—head and neck surgery (OHNS). Clinical practice in OHNS is rich with image, audio, video, genetic, and neurophysiologic semi-structured data that offer abundant opportunities for AI analysis. Indeed, an increasing number of publications have explored proof-of-concept applications of AI in OHNS^1–3^.

Despite these opportunities, otolaryngologists today use a small number of AI tools for clinical work, most of which are general tools not specific to OHNS (e.g., AI-powered voice dictation and ambient scribing of clinic notes). Part of the reason for this practice is that there are few OHNS-specific AI tools that are ready for clinical deployment. As of August 7, 2024, among 950 FDA-approved AI/machine learning-enabled medical devices^4^ only two were developed specifically for OHNS^5,6^. Clearly, there is a discrepancy between the opportunity for developing clinically useful AI tools in OHNS and the realization of this opportunity; this trend has been more broadly termed the “AI chasm” in healthcare^7^.

Clinical studies are important to translate AI tools by validating their utility and readiness for deployment in the healthcare setting. We hypothesize that clinical validation is an understudied area of research in development of AI applications in OHNS. If true, the finding would identify an area needing attention in clinically targeted AI research. To evaluate this hypothesis, we conducted a scoping review of deep learning research in OHNS with attention to the stages of AI model development (i.e., proof-of-concept, offline validation, clinical validation phase) and approaches for model validation used.

## Results

**Fig. 1:**
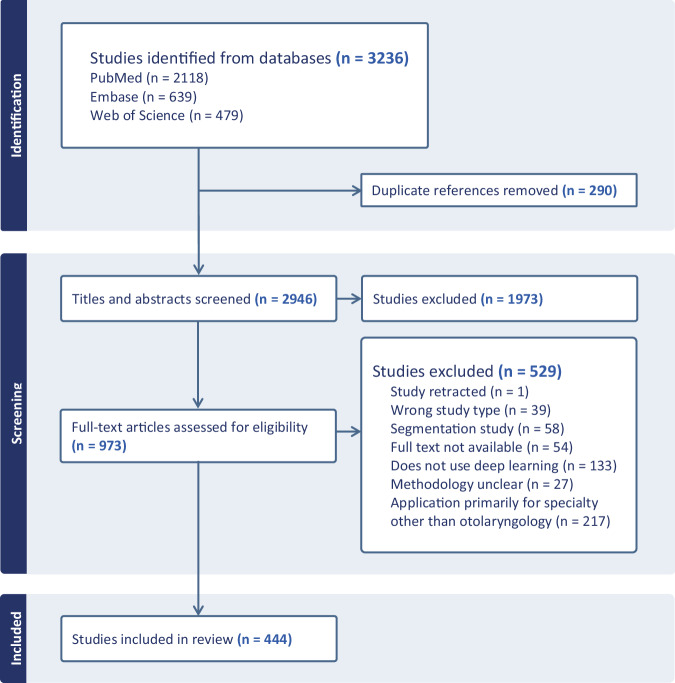
PRISMA flow diagram. A total of 444 studies were included in the scoping review.

We identified 3236 records in databases searches. After de-duplication, we screened 2946 abstracts and titles and 973 full-text articles. Ultimately, 444 publications met inclusion criteria (Fig. 1; Supplementary Data). Hereafter the included studies are referred to as the deep learning publications in OHNS and the term AI specifically refers to deep learning methods.

**Fig. 2:**
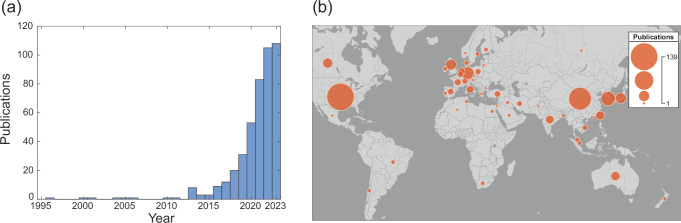
Temporal and geospatial distribution of deep learning publications in OHNS. **a** Publications per year on deep learning applications in OHNS, as of October 25, 2023. **b** Geographic bubble chart of country affiliations of publications. For each country, the circled area indicates the number of publications with at least one author affiliated with an institution in that country. The map was created using Natural Earth.

The temporal and geospatial distribution of deep learning publications in OHNS are shown in Fig. 2. In a 10-year period between 2012 and 2022, there was an exponential increase in publications per year from 0 publications in 2012 to 105 publications in 2022, the last full calendar year included in this review (Fig. 2a). Author affiliations spanned 48 countries across six continents, demonstrating the global scale of AI research (Fig. 2b). The countries with the highest numbers of publications were the United States (139 publications), China (95 publications), and South Korea (38 publications) (Supplementary Fig. 1).

**Fig. 3:**
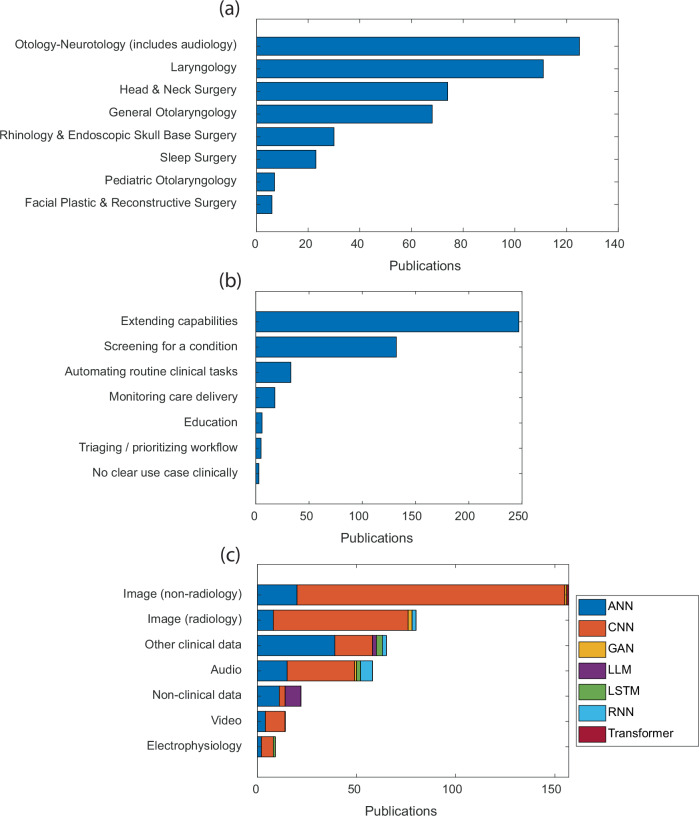
Sub-specialty, application type, and data type distributions of deep learning research in OHNS. **a** Primary OHNS sub-specialties of AI applications in publications. **b** Application type of deep learning models in publications. **c** Data type of inputs to deep learning models in publications, sub-categorized by the types of deep learning models used to analyze the data. Abbreviations: ANN artificial neural network, CNN convolutional neural network, GAN generative adversarial network, LLM large language model, LSTM long-short term memory, RNN recurrent neural network.

Additional descriptive characteristics of deep learning publications in OHNS are shown in Fig. 3. Deep learning research spanned all OHNS subspecialties with the highest number of publications in otology and neurotology (including audiology) (28%) (Fig. 3a). The most common goal of AI applications was to extend the capabilities of health care providers (56%), and the second most common goal was to screen for medical conditions (30%) (Fig. 3b). Non-radiology (36%) and radiology (19%) images were the most common data types that AI models analyzed. The most common non-radiology images analyzed were otoscopy, laryngoscopy, clinical photography, histology, hyperspectral, and nasal endoscopy images (Supplementary Table 1 and Supplementary Fig. 2). Convolutional neural network (CNN) models were the most used deep learning model type for analyzing image, audio, video, and electrophysiology data (Fig. 3c).

**Fig. 4:**
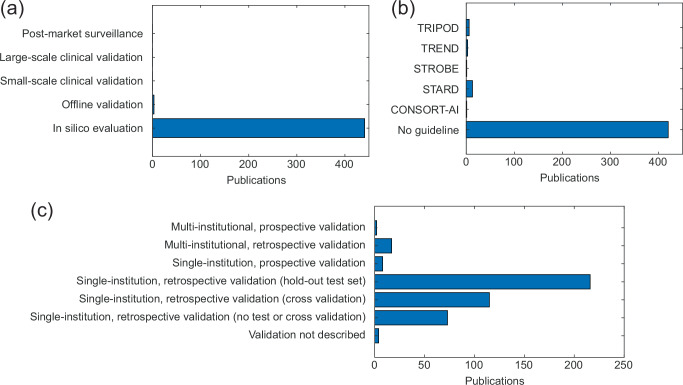
Stages of AI model development, reporting guidelines, and validation methods used by AI research in OHNS. **a** Stages of AI model development in publications. **b** Reporting guidelines used by publications. **c** Evaluation methods used by publications. Abbreviations: TRIPOD Transparent reporting of a multivariable prediction model for individual prognosis or diagnosis, TREND Transparent Reporting of Evaluations with Nonrandomized Designs, STROBE Strengthening the Reporting of Observational Studies in Epidemiology, STARD Standards for Reporting of Diagnostic Accuracy Studies, CONSORT-AI Consolidated Standards of Reporting Trials–Artificial Intelligence.

The stages of AI models, adherence to reporting guidelines, and validation methods in included publications are shown in Fig. 4. The stages for AI in healthcare provide a useful framework for assessing the maturity of validation studies of an AI tool and its readiness for clinical deployment^8^. Nearly all studies (99.3%) were in the in silico proof-of-concept stage. Three (0.7%) studies moved beyond in silico development to offline validation (Fig. 4a). These three studies assessed speech denoising^9^, visual speech recognition^10^, and speaker separation^11^ AI models in human subjects in an experimental setting. Strikingly, there were zero (0%) clinical validation studies among the 444 deep learning publications in OHNS.

Reporting guidelines involve recommendations, often in the form of checklists, of essential information to include in the dissemination of research results. Reporting guidelines are particularly important for AI research in healthcare, a rapidly evolving field, and can improve reproducibility, transparency, and standardization. Despite this requirement, reporting guidelines were used infrequently by only 24 studies (5.4%) (Fig. 4b). Reporting guidelines used were, in decreasing order, the Standards for Reporting of Diagnostic Accuracy Studies (STARD)^12^ (2.9%), Transparent reporting of a multivariable prediction model for individual prognosis or diagnosis (TRIPOD)^13^ (1.3%), Transparent Reporting of Evaluations with Nonrandomized Designs (TREND)^14^ (0.7%), Strengthening the Reporting of Observational Studies in Epidemiology (STROBE)^15^ (0.2%); and Consolidated Standards of Reporting Trials–Artificial Intelligence (CONSORT-AI)^16^ (0.2%).

Single-institution and retrospective validation methods were most used (Fig. 4c), with only 10 studies (2.3%) using prospective evaluation for either single-institution (1.8%) or multi-institution (0.5%) datasets. Notably, 4 studies (0.9%) did not describe their validation method, and 73 studies (16%) reported retrospective validation results with neither an independent test set nor cross-validation, which is noteworthy because it limits assessment of a model’s generalizability^17^.

Explainability of AI models is important for trust and reproducibility. Forty-one studies (9.2%) described use of a method to attempt to explain the AI model. Two of the most used methods for explainability were Gradient-weighted Class Activation Mapping (Grad-CAM) (3%)^18^ and Class Activation Maps (CAM)^19^ (2%).

## Discussion

Despite the promise of AI technology to transform precision medicine and healthcare, there has been a chasm between the potential benefit of AI tools and their variable performance when deployed clinically^7^. We demonstrate that the AI chasm is particularly deep in the field of OHNS, as there is a striking absence of clinical validation studies among over 440 deep learning publications in OHNS between 1996–2023. Increasing clinical validation studies, as well as moving beyond proof-of-concept studies, is important to advance the development of clinically useful AI applications in OHNS. To the best of our knowledge, this is the first scoping review of deep learning applications that addresses the entire field of OHNS.

The AI chasm in OHNS mirrors challenges in translating AI technology to healthcare more broadly. Clinical validation studies have demonstrated mixed performance of AI models for sepsis prediction using electronic health record data^20^, real-time diabetic retinopathy screening^21^, and chest x-ray screening^22^. Even if AI models were highly accurate and surpassed human diagnostic capabilities, they would not necessarily enable better care because that would also depend on the healthcare system’s ability to take appropriate actions based on the AI model’s output^23^. Three practical aspects of model design—actionability, safety, and utility—have been proposed to help bridge the AI chasm^24^. Additional issues that are likely to contribute to the AI chasm include the substantial cost for developing, implementing, and monitoring AI models and liability. Healthcare is a highly regulated industry, and compliance with regulations contributes to the cost and challenges of deploying and clinically validating AI models^25^.

Our review informs the following suggestions for advancing the development of clinically useful AI models in OHNS. First, we suggest increasing effort and funding for AI research addressing low complexity, low-risk tasks because these applications have the potential to provide real world benefit sooner and in a more clearly defined way than higher complexity, higher risk tasks. This could involve refocusing AI research on non-diagnostic applications in OHNS, such as automating routine tasks and triaging workflows, rather than diagnostic applications, such as extending capabilities of healthcare providers and screening for medical conditions which together constitute 86% of deep learning publications in OHNS (Fig. 3b). Even automating routine tasks carries risk, however, as major health insurers have faced class action lawsuits for using AI models to automate insurance preauthorization.

Second, we suggest adherence to reporting guidelines for AI prediction tools. These guidelines are increasing in number^26^ and provide frameworks for standardizing reporting and transparency that can improve the fairness, usefulness, and reliability of AI models^7,27^. Guidelines can help researchers anticipate challenges for clinical translation of an AI model prior to its design and development and improve the quality and reporting of validation methods, which were potentially inadequate (lacking a hold-out test set or use of cross-validation) in 77 studies (17%) (Fig. 4c). The fact that only 5.4% of included studies used a reporting guideline highlights the opportunity for improvement in this regard (Fig. 4b). There remains a challenge in deciding which among the several reporting guidelines to use, and more work is needed to shape understanding of what an accepted standard reporting guideline should be.

Third, we suggest that researchers prioritize clinical validation of AI applications. From our experience in conducting proof-of-concept AI research in OHNS^28–35^, we recognize the hurdles to translating AI research. One perceived challenge is the assumption that external validation on test datasets from (often multiple) outside institutions is required to assess an AI model’s generalizability. Recurring local validation has been proposed as a potentially advantageous alternative to external validation^36^. Adopting a standard of site-specific, local validation could encourage researchers to pilot small-scale clinical validation studies at their institution as part of an iterative process to improve clinical validation and lower the barrier to initiating clinical studies. At the same time, it may protect against model drift^36,37^. To ensure that validation still includes a diverse patient population, we would still encourage the use of multi-institutional data for initial model training. The need to validate AI models at scale will reinforce previous efforts to create multicenter data-sharing collaborations in OHNS^38^. The use of frameworks such as federated^39^ and swarm learning^40^ can help maintain the confidentiality of patient data and address data-sharing barriers for building large, representative datasets and accelerating model development and clinical validation.

Finally, we suggest careful attention to the accuracy of ground truth labels in OHNS datasets to promote the success of predictive AI models. Clinical diagnoses do not always serve as the most accurate or precise “ground truth” labels for AI training datasets. For example, in laryngology-based AI research related to voice disorders, participants categorized with “hyperfunctional” voice disorders may include those in the early phases of vocal pathophysiology prior to lesion formation (i.e., vocal fold edema), or those who have already developed nodules, polyps, or contact ulcers^32^. Superordinate categorization of pathology may obscure the uniqueness of more refined diagnostic categories when applying AI models for predictive objectives.

Though interpretability can influence trust, we do not suggest that future studies must attempt to explain their AI models, a process that was done in only 41 studies (9.2%). The requirement that AI models be explainable to be safely used in medicine is under debate^41,42^, though lack of explainability likely contributes to distrust of AI models.

This study has several limitations. First, the search terms were initially designed to capture studies focused on developing rather than evaluating existing deep learning applications in OHNS. This resulted in the omission of some peer-reviewed research letters evaluating the application of Chat-GPT to OHNS^43^. Second, although our team of seven reviewers independently screened and extracted data from included studies, our process relied on adjudication by a single reviewer with expertise in AI and clinical OHNS. This review process could be improved in the future, as demonstrated by the fair interrater agreement of 73% (Cohen’s Kappa 0.39) for title and abstract screening. Third, some parts of the data extraction relied on subjective judgment, such as assessing adherence to reporting guidelines, stages of AI model development, and validation methods. Use of more explicit instructions in the data extraction form, for example, listing all relevant reporting guidelines, could make the data extraction more consistent. Fourth, aside from publication years, the data were analyzed collectively across all years of publication, which may inadvertently hide trends in deep learning research across different time periods. Fifth, this review’s database searches were conducted in October 2023 and may miss important studies published since then. Future studies should update this review to include new studies and monitor the rapid progress of AI research in OHNS. Such updates will be important to surveil trends in large language model applications in OHNS^44,45^ and multimodal AI^46^ which offer cutting-edge approaches for developing holistic and clinically-relevant models. Sixth, while this review conducted a broad survey of AI applications in OHNS, further descriptions of individual applications can be found in recent scoping reviews of AI in audiology^47^, laryngology^3^, and other OHNS sub-specialties.

The framework for “translational research” in AI applications for OHNS is still being established. This review identifies a clear gap in the AI literature in OHNS for clinical validation studies. Our recommendations to help fill this gap include focusing on low complexity and low risk tasks, adhering to reporting guidelines, and prioritizing clinical translation while keeping rigorous standards of diversity in our datasets. If successful, clinical translation of AI technology in OHNS might serve as a blueprint for the broader healthcare community to cross the AI chasm.

## Methods

### Search strategy and inclusion criteria

We conducted a scoping review of the literature on deep learning applications in OHNS following the PRISMA Extension for Scoping Reviews guidelines^48^ (Supplementary Table 2). Our search strategy was developed in collaboration with a research librarian (Supplementary Table 3). Search terms were chosen to capture studies that developed or evaluated deep learning models that were intended to be primarily applied to the field of OHNS. We executed the queries from October 16th to October 25th, 2023, to search the MEDLINE, EMBASE, and Web of Science databases.

Inclusion criteria were the development or evaluation of a deep learning model primarily intended to be applied to the field of OHNS. Deep learning models included neural networks (e.g., artificial, convolutional, recurrent, long-short term memory, generative adversarial) and large language models. Three categories of studies fell outside the inclusion criteria and, therefore, were excluded. First, studies primarily targeting another specialty (e.g., prediction of apnea-hypopnea index for sleep medicine or nasopharyngeal cancer patient survival after radiation treatment for radiation oncology) were excluded. The primary specialty targeted by an AI application was determined based on which physician specialty the AI application was intended to be used by clinically. For example, OHNS applications included analysis of radiology images to aid clinical decision making by otolaryngologists (e.g., prediction of inverted papilloma malignant transformation on magnetic resonance imaging scans^28^). Second, studies of machine learning methods (e.g., multilayer perceptron, logistic regression, and support vector machines) that did not involve deep learning were excluded. Third, general speech recognition tasks that did not directly relate to otolaryngology were excluded, even if these tasks involved the analysis of voice data (e.g., general-purpose speech to text prediction models). Finally, only peer-reviewed, original research articles published in the English language that had retrievable full texts were considered.

### Screening process

A team of seven reviewers (G.S.L., S.F., M.C.L., S.P., J.H., S.K., and M.S.,) conducted the literature review using Covidence, a collaborative web-based platform^49^. References found by the search strategy were de-duplicated and screened using the inclusion criteria, first by title and abstract, and then by full text. Full texts were reviewed by one reviewer and checked by a second reviewer. Discrepancies between reviewers were decided by an adjudicator with expertise in deep learning and otolaryngology (G.S.L.). Studies that passed the full-text screening phase were included in the review.

### Data extraction and analysis

We extracted the following data from included studies: article information (e.g., year of publication, countries of authors’ affiliated institutions), deep learning method, application (i.e., the goal of the application and target sub-specialty within OHNS), input data, method for model validation, stage of model development, use of reporting guidelines, and attempts to explain the model. Our data extraction form is available in Supplementary Table 4. If a study investigated multiple deep learning methods and data types, the primary model and data type in the study were chosen. We categorized model validation methods according to whether data were collected from single versus multiple institutions, obtained prospectively versus retrospectively, and/or partitioned into a hold-out test dataset or cross-validation folds. Omission of the use of either an independent test dataset or cross-validation limits assessment of the generalization performance of the model with future, unseen data^17^. Evaluation of validation methods erred on the more robust methodology in cases of uncertainty to provide an upper bound on the quality of validation. For example, a study that reported “test” results but did not explicitly describe the use of a hold-out test dataset was presumed to have used one.

We categorized the stages of AI model development according to the stages for AI in healthcare described in the DECIDE-AI reporting guidelines^8^: in silico evaluation (i.e., proof of concept), offline validation (i.e., silent/shadow evaluation), small-scale clinical validation, large-scale clinical validation, and post-market surveillance. For consistency, we considered in silico evaluation as application of an AI model to prepared data in a context removed from the context of the intended use (e.g., assessment of a speech denoising algorithm on audio recordings saved on a computer); offline validation as application to prospective data in a context similar to the intended use (e.g., assessment of a speech denoising algorithm in cochlear implant subjects in the laboratory); and clinical validation as application in the context of the intended use (e.g., assessment of a speech denoising algorithm in cochlear implant subjects outside the laboratory). Disagreements during data extraction were decided by the adjudicator (G.S.L.).

## Supplementary information

Supplementary Information

Supplementary Data

## Data Availability

The complete dataset of studies included in the scoping review is provided in the Supplementary Data.
