# Supplementary material for: Scoping review of deep learning research illuminates artificial intelligence chasm in otolaryngology-head and neck surgery
Source: NPJ Digit Med. 2025 May 10;8:265. doi: 10.1038/s41746-025-01693-0 (PMC12064819; doi:10.1038/s41746-025-01693-0)
Supplement: Supplementary file 1 — Supplementary Information [file 41746_2025_1693_MOESM1_ESM.pdf]

Supplementary Information

Supplementary Table 1. List of non-radiology image data types analyzed in included studies.

**Non-radiology image type**	**Publications**
Otoscopy images	37
Laryngoscopy images	35
Clinical photographs	20
Histology	15
Hyperspectral imaging	11
Nasal endoscopic images	9
Pharyngeal endoscopy	4
VFSS	4
Raman spectroscopy	3
Multispectral imaging	2
Autofluorescence spectroscopy	1
Chromogenic agar plate images	1
DISE	1
EEG	1
FEES	1
Fluorescence lifetime imaging	1
Infrared thermal imaging	1
Narrow band imaging	1
Optical coherence tomography images	1
Otoscopy videos	1
Pharyngeal high-resolution manometry with impedance	1
Surgical video	1
Thyroid endoscopic images	1
VNG	1

Supplementary Table 2. Preferred Reporting Items for Systematic reviews and Meta-Analyses extension for Scoping Reviews (PRISMA-ScR) Checklist

SECTION	ITEM	PRISMA-ScR CHECKLIST ITEM	REPORTED ON PAGE #
**TITLE**			
Title	1	Identify the report as a scoping review.	1
**ABSTRACT**			
Structured summary	2	Provide a structured summary that includes (as applicable): background, objectives, eligibility criteria, sources of evidence, charting methods, results, and conclusions that relate to the review questions and objectives.	2
**INTRODUCTION**			
Rationale	3	Describe the rationale for the review in the context of what is already known. Explain why the review questions/objectives lend themselves to a scoping review approach.	3
Objectives	4	Provide an explicit statement of the questions and objectives being addressed with reference to their key elements (e.g., population or participants, concepts, and context) or other relevant key elements used to conceptualize the review questions and/or objectives.	3
**METHODS**			
Protocol and registration	5	Indicate whether a review protocol exists; state if and where it can be accessed (e.g., a Web address); and if available, provide registration information, including the registration number.	10
Eligibility criteria	6	Specify characteristics of the sources of evidence used as eligibility criteria (e.g., years considered, language, and publication status), and provide a rationale.	10
Information sources*	7	Describe all information sources in the search (e.g., databases with dates of coverage and contact with authors to identify additional sources), as well as the date the most recent search was executed.	10
Search	8	Present the full electronic search strategy for at least 1 database, including any limits used, such that it could be repeated.	18

SECTION	ITEM	PRISMA-ScR CHECKLIST ITEM	REPORTED ON PAGE #
Selection of sources of evidence†	9	State the process for selecting sources of evidence (i.e., screening and eligibility) included in the scoping review.	10
Data charting process‡	10	Describe the methods of charting data from the included sources of evidence (e.g., calibrated forms or forms that have been tested by the team before their use, and whether data charting was done independently or in duplicate) and any processes for obtaining and confirming data from investigators.	11
Data items	11	List and define all variables for which data were sought and any assumptions and simplifications made.	11
Critical appraisal of individual sources of evidence§	12	If done, provide a rationale for conducting a critical appraisal of included sources of evidence; describe the methods used and how this information was used in any data synthesis (if appropriate).	n/a
Synthesis of results	13	Describe the methods of handling and summarizing the data that were charted.	12
**RESULTS**			
Selection of sources of evidence	14	Give numbers of sources of evidence screened, assessed for eligibility, and included in the review, with reasons for exclusions at each stage, ideally using a flow diagram.	4
Characteristics of sources of evidence	15	For each source of evidence, present characteristics for which data were charted and provide the citations.	Supplementary Data
Critical appraisal within sources of evidence	16	If done, present data on critical appraisal of included sources of evidence (see item 12).	n/a
Results of individual sources of evidence	17	For each included source of evidence, present the relevant data that were charted that relate to the review questions and objectives.	Supplementary Data
Synthesis of results	18	Summarize and/or present the charting results as they relate to the review questions and objectives.	4-6
**DISCUSSION**			
Summary of evidence	19	Summarize the main results (including an overview of concepts, themes, and types of evidence available), link to the review questions and objectives, and consider the relevance to key groups.	6

SECTION	ITEM	PRISMA-ScR CHECKLIST ITEM	REPORTED ON PAGE #
Limitations	20	Discuss the limitations of the scoping review process.	9
Conclusions	21	Provide a general interpretation of the results with respect to the review questions and objectives, as well as potential implications and/or next steps.	10
**FUNDING**			
Funding	22	Describe sources of funding for the included sources of evidence, as well as sources of funding for the scoping review. Describe the role of the funders of the scoping review.	13

JBI = Joanna Briggs Institute; PRISMA-ScR = Preferred Reporting Items for Systematic reviews and Meta-Analyses extension for Scoping Reviews.
* Where *sources of evidence* (see second footnote) are compiled from, such as bibliographic databases, social media platforms, and Web sites.
† A more inclusive/heterogeneous term used to account for the different types of evidence or data sources (e.g., quantitative and/or qualitative research, expert opinion, and policy documents) that may be eligible in a scoping review as opposed to only studies. This is not to be confused with *information sources* (see first footnote).
‡ The frameworks by Arksey and O'Malley (6) and Levac and colleagues (7) and the JBI guidance (4, 5) refer to the process of data extraction in a scoping review as data charting.
§ The process of systematically examining research evidence to assess its validity, results, and relevance before using it to inform a decision. This term is used for items 12 and 19 instead of "risk of bias" (which is more applicable to systematic reviews of interventions) to include and acknowledge the various sources of evidence that may be used in a scoping review (e.g., quantitative and/or qualitative research, expert opinion, and policy document).

From: Tricco AC, Lillie E, Zarin W, O'Brien KK, Colquhoun H, Levac D, et al. PRISMA Extension for Scoping Reviews (PRISMAScR): Checklist and Explanation. Ann Intern Med. 2018;169:467–473. doi: 10.7326/M18-0850.

Supplementary Table 3. Search strategy for PubMed, EMBASE, and Web of Science databases.

Database	Search terms
PubMed	("artificial intelligence" [tw] OR "artificial neural network*" [tw] OR "convolutional neural network*" [tw] OR "deep learning" [tw] OR "deep neural network*" [tw] OR "artificial intelligence" [mesh] OR "neural networks, computer" [mesh] OR "deep learning" [mesh]) AND ("otolaryngology" OR "Otorhinolaryngologic Surgical Procedures"[Mesh] OR "Otorhinolaryngologic Diseases"[Mesh] OR "Otolaryngology"[Mesh] OR otorhinolaryngology* [tw] OR otolaryng* [tw] OR "ear nose and throat" [tw] OR ent [tw] OR "otolaryngologists" [tw]) AND English [lang]
EMBASE	('artificial intelligence'/exp OR 'neural networks'/exp OR 'deep learning'/exp OR 'artificial neural network'/exp OR "artificial intelligence":ti,ab,kw OR "artificial neural network":ti,ab,kw OR "convolutional neural network":ti,ab,kw OR "deep learning":ti,ab,kw OR "deep neural network*":ti,ab,kw) AND ("otolaryngology":ti,ab,kw OR 'ear nose throat surgery'/exp OR 'otorhinolaryngology'/exp OR 'ear nose throat disease'/exp OR otorhinolaryngology*:ti,ab,kw OR otolaryng*:ti,ab,kw OR "ear nose and throat":ti,ab,kw OR ent:ti,ab,kw OR "otolaryngologists":ti,ab,kw) AND [english]/lim AND ([embase]/lim NOT ([embase]/lim AND [medline]/lim) OR [preprint]/lim)
Web of Science	TS=("artificial intelligence" OR "artificial neural network" OR "convolutional neural network" OR "deep learning" OR "deep neural network*") AND TS=(otorhinolaryngology* OR otolaryng* OR "ear nose and throat" OR ent OR "otolaryngologists")

Supplementary Table 4. Data extraction form.

**Article information**	
Study ID	
Title of Article	
Last name of first Author	
Year of Publication	
Country in which the study was conducted	
Possible conflicts of interest	
**Deep learning methods and data**	
Goal of deep learning application	- Screening for a condition<br>- Triaging / prioritizing workflow<br>- Automating routine clinical tasks<br>- Monitoring care delivery / double-checking<br>- Extending MD capabilities in diagnosis or treatment on par or exceeding subspecialists<br>- Education<br>- No clear use case clinically<br>- Other
Domain of Otolaryngology	- General Otolaryngology<br>- Pediatric Otolaryngology<br>- Facial Plastic & Reconstructive Surgery<br>- Head & Neck Surgery<br>- Laryngology<br>- Otology-Neurotology (includes audiology)<br>- Rhinology & Endoscopic Skull Base Surgery<br>- Sleep Surgery<br>- Other
Deep learning model category	- (Artificial) neural network (ANN)<br>- Convolutional neural network (CNN)<br>- Recurrent neural network (RNN)<br>- Long Short Term Memory Networks (LSTMs)<br>- Generative Adversarial Networks (LSTMs)<br>- Large language model (LLM), e.g.,Chat-GPT<br>- Transformer model (other than LLMs)<br>- Other
Data analyzed	

Date Category	- Image - radiology<br>- Image - non-radiology<br>- Audio<br>- EHR / clinical data (other than image or audio)<br>- Other
Stage of AI model for healthcare use	- In silico evaluation (proof of concept)<br>- Offline validation (silent/shadow evaluation)<br>- Safety/utility, small-scale (early live clinical evaluation)<br>- Safety/effectiveness, large-scale (comparative prospective evaluation)<br>- Post-market surveillance
Was a reporting guideline followed in describing the study's evaluation of its AI-based decision support system?	- Yes<br>- No
If a reporting guidelines was used, what was its name?	
How was the AI model evaluated?	- Multi-institutional, prospective validation<br>- Multi-institutional, retrospective validation<br>- Single-institutional, prospective validation<br>- Single-institutional, retrospective validation using held-out test dataset<br>- Single-institutional, retrospective validation using cross-validation on training/development dataset<br>- Single-institutional, retrospective validation only (no hold-out test set or cross validation, includes concurrent validation)<br>- No validation was performed
Were methods used to attempt to explain the AI model?	- Yes<br>- No
If so, describe what attempt was made to explain model.	

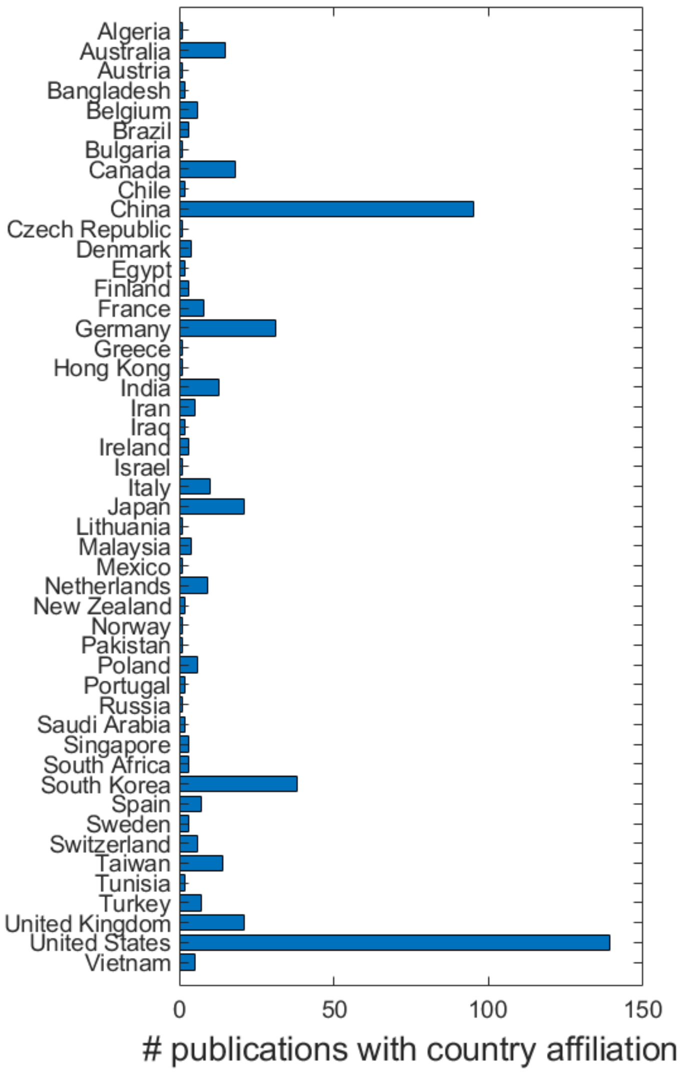

Supplementary Figure 1. Country affiliations of authors of deep learning publications included in the scoping review. This bar plot enumerates the data shown in **Figure 2b**.

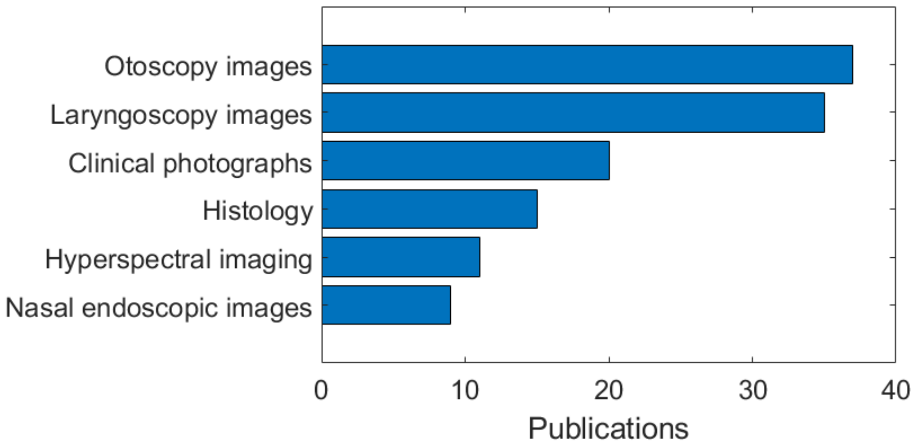

Supplementary Figure 2. Types of non-radiology images analyzed by deep learning models in studies included in the scoping review. Only image data types with more than 5 publication counts are shown.

Supplementary Data no. 1

AI_OHNS_scoping_review_includedstudies_submission.xlsx - Excel file with list of included studies.
